# Childhood Adversity Predicts Later-life Memory Lapses, Irritation and Interference

**DOI:** 10.1093/geroni/igaf122.3557

**Published:** 2025-12-31

**Authors:** Negar Mokhtari, Kat Barrett, Heejung Jang, Jody Nicholson, Lulu Hottinger

**Affiliations:** Clemson University, Clemson, South Carolina, United States; Clemson University, Clemson, South Carolina, United States; The Pennsylvania State University, Asian/Pacific Islander, Pennsylvania, United States; Clemson University, Clemson, South Carolina, United States; Clemson University, Clemson, South Carolina, United States

## Abstract

This study investigated relationships between childhood adversity (CA) domains—abuse (physical, sexual, emotional) and neglect (physical, emotional) — daily prospective memory (PM) and retrospective memory (RM) lapse frequency, and memory lapse (ML) related irritation and interference in middle-to-older adults. Participants (N = 1,236; Mage=62.5±10.2) disclosed histories of CA in the 2013-2014 wave of the Midlife in the United States longitudinal study and completed eight nightly reports on MLs in the 2017-2019 wave. In univariate analyses, all five CA domains were significantly associated with PM (p<.001 - .007). Additionally, emotional and physical neglect were univariately significant for RM (p<.001). In the full ML model, no CA domains significantly predicted RM, and only emotional neglect (p=.010) significantly predicted PM. Emotional abuse (p=.039), sexual abuse (p=.032) and physical neglect (p=.007) predicted PM-related irritation, while sexual (p=.044), physical abuse (p=.045) and physical neglect (p=.001) predicted PM-related interference. Physical neglect (p =.035) also remained significant in the full PM model. For RM-related irritation and interference, all CA domains were significant univariately (p<.001). In the full RM model, physical abuse predicted irritation (p =.008), while physical (p=.003) and sexual (p=.017) abuse predicted interference. Overall, every domain of CA predicted daily PM lapses (p = <.001 - .007) and even more strongly predicted RM lapse-related irritation and interference (p = < .001). Findings suggest a history of CA increases the severity of RM lapse-related interference and irritation more than PM lapse frequency in later life. Future person-centered analyses are needed to assess the potential for shared variance between CA domains resulting in suppression effects.

